# Dramatic band gap reduction incurred by dopant coordination rearrangement in Co-doped nanocrystals of CeO_2_

**DOI:** 10.1038/s41598-017-05046-0

**Published:** 2017-07-05

**Authors:** T. S. Wu, Y. W. Chen, S. C. Weng, C. N. Lin, C. H. Lai, Y. J. Huang, H. T. Jeng, S. L. Chang, Y. L. Soo

**Affiliations:** 1 Department of Physics, National Tsing Hua University, Hsinchu, Taiwan; 2 National Synchrotron Radiation Research Center, Hsinchu, Taiwan; 3 Department of Biomedical Engineering and Environmental Sciences, National Tsing Hua University, Hsinchu, Taiwan; 4 Department of Materials Science and Engineering, National Tsing Hua University, Hsinchu, Taiwan; 5 Institute of Physics, Academia Sinica, Taipei, Taiwan

## Abstract

A dramatic band gap narrowing of 1.61 eV has been observed in Co-doped nanocrystals of CeO_2_ (ceria), as a result of thermal annealing, without changing the ceria crystal structure and the Co concentration. As demonstrated by x-ray absorption fine structures, thermal annealing incurs an oxygen coordination rearrangement around Co atoms from an octahedral coordination to a square-planar coordination. First principle calculation using density functional theory reveals two stable oxygen coordination types surrounding Co, consistent with the experimental observation. The band gap values calculated for the two stable coordination types differ dramatically, reproducing the experimentally observed band gap narrowing. These prominent effects due to local structure rearrangement around dopant atoms can lead to unprecedented methods for band gap engineering in doped nanocrystal oxides.

## Introduction

Ceria based catalysts have attracted considerable research interest due to their important applications in energy and environmental technologies. The catalytic activities quantified by CO oxidation for the ceria catalysts have been reported to improve after metal ion doping^1–3^. Theoretical works aiming to establish models for their catalytic mechanism and activity improvements have also been extensively performed^4–6^. It is well-known that increased ionic conductivity and electronic conductivity due to oxygen vacancies arising from anion deficiency can enhance the catalytic activity in thermally annealed non-stoichiometric ceria^7^. On the other hand, thermal annealing was reported to release strains and thus change the band gap in many solid-state materials^8–10^. It is conceivable that reduction of the band gap of ceria can increase the efficiency of charge transfer required in the catalytic processes and therefore also contribute to enhance the catalyst’s activity. In this paper, we demonstrate a newly observed bistability of local oxygen coordination around Co dopant atoms. Transition between the two stable oxygen coordination types can be incurred by thermal annealing. In contrast to the strain release mechanism, such oxygen-coordination-type transition of Co dopant can give rise to much more dramatic band-gap variations.

**Figure 1:**
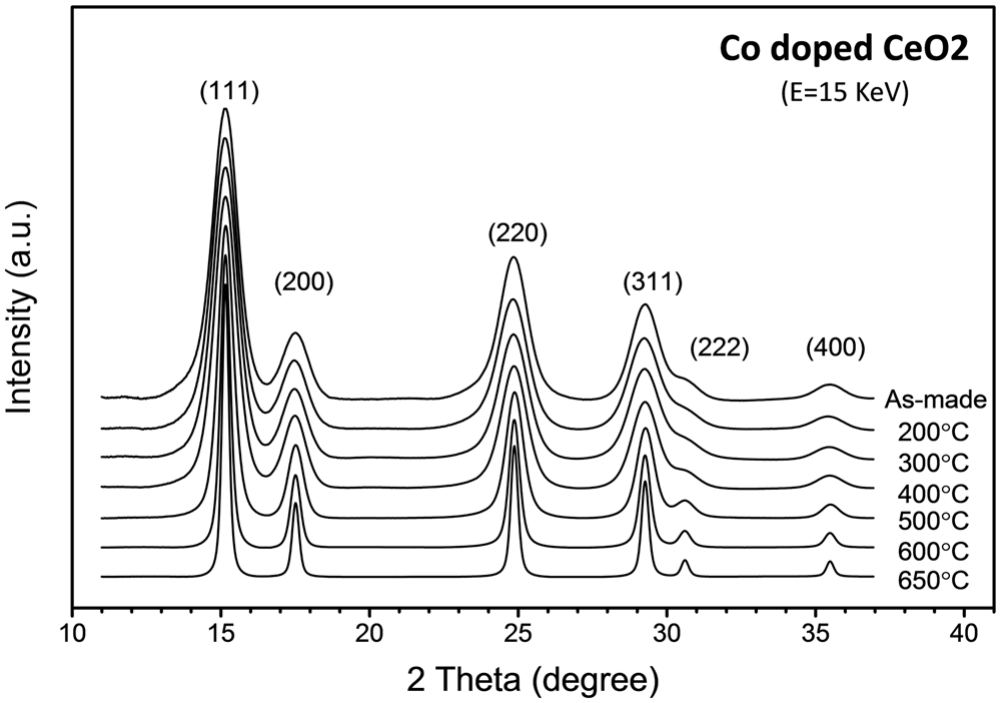
X-ray powder diffraction patterns of Co-doped CeO_2_ samples annealed at different temperatures. Curves have been shifted vertically for the sake of clarity.

Nanocrystal samples of Co-doped CeO2 synthesized by using a polyol method^11^ was annealed at temperatures 200, 300, 400, 500, 600, and 650 °C. The Co concentration of the samples was determined to be 4.3 at. % by using inductively coupled plasma mass spectrometry (ICPMS). As shown in Fig. 1, the synchrotron-based x-ray powder diffraction (XRD) patterns for all samples match well with that of cubic CeO_2_ at the (111), (200), (220), (311), (222) and (400) Bragg peaks indicating that the crystal structure remains largely unchanged after annealing. The crystallite size determined by the Scherrer equation increases from 4.0 nm in the as-made sample to 4.0, 4.2, 4.6, 6.7, 10.6, and 15.3 nm in the 200 °C-, 300 °C-, 400 °C-, 500 °C-, 600 °C-, and 650 °C-annealed sample while the corresponding lattice parameter changes from 5.4216 Å to 5.4220, 5.4222, 5.4197, 5.4177, 5.4173, and 5.4169 Å, respectively. The decreasing trend of lattice parameter with increasing crystallite size is consistent with previous reports on CeO_2_ nanoparticle^12^.

**Figure 2:**
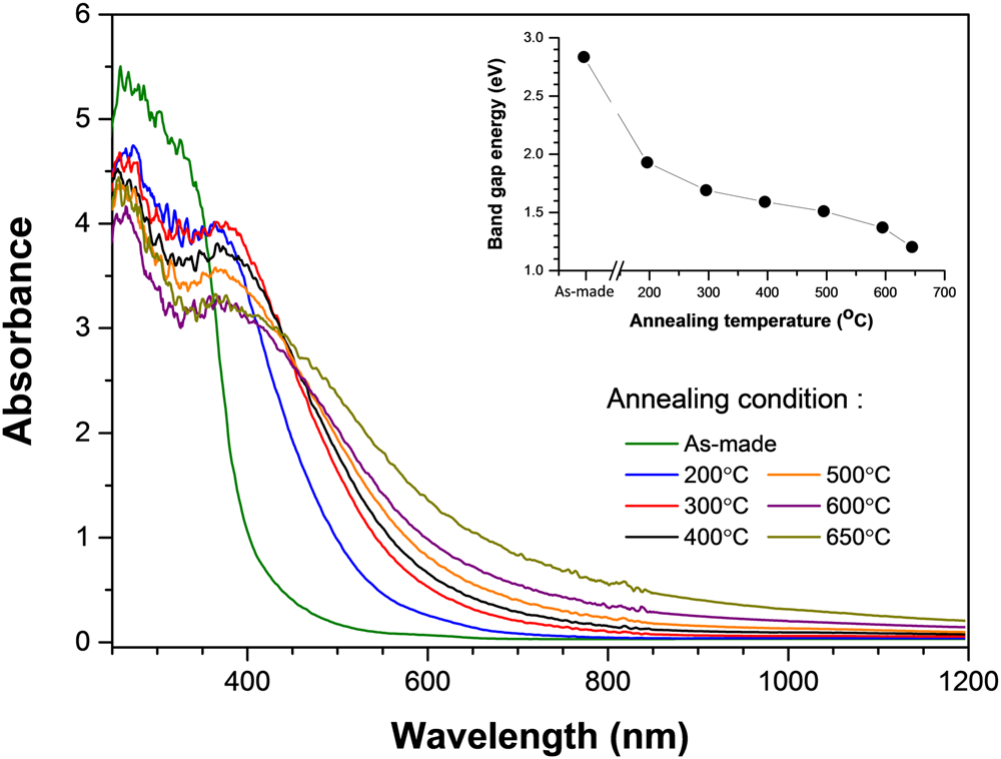
UV–vis diffuse reflectance spectra for Co-doped CeO_2_ samples. Inset: A plot of band gap energy vs. annealing temperature.

However, dramatic changes in band gap width were observed in the annealed samples from UV-vis diffuse reflectance spectra as shown in Fig. 2. To extract the band gap values from the experimental data, the Kubelka-Munk^13^ function and Tauc’s plots^14^ were used. The band gap energies, determined by the intersection of the horizontal axis and the tangent line to the absorption curve at the inflection point, are 2.80 eV, 1.94 eV, 1.71 eV, 1.60 eV, 1.48 eV, 1.36 eV and 1.19 eV, for the as-made, 200, 300, 400, 500, 600 and 650 °C annealed sample, respectively. The band gap energy of the sample shows a drastic decrease of 1.61 eV after thermal annealing at 650 °C. It is worth noting that the band gap width of the as-made sample of Co-doped CeO_2_ nanocrystals is roughly the same as that of a pure CeO_2_ nanocrystal, 2.80 eV measured previously^11^. The effect of Co dopant atoms on band-gap engineering of ceria appears to be negligible compared to that of thermal annealing in the present work. Also, the band gap variation due to crystallite-size-related quantum confinement effect was reported to be only around 0.2 eV^15, 16^.

**Figure 3:**
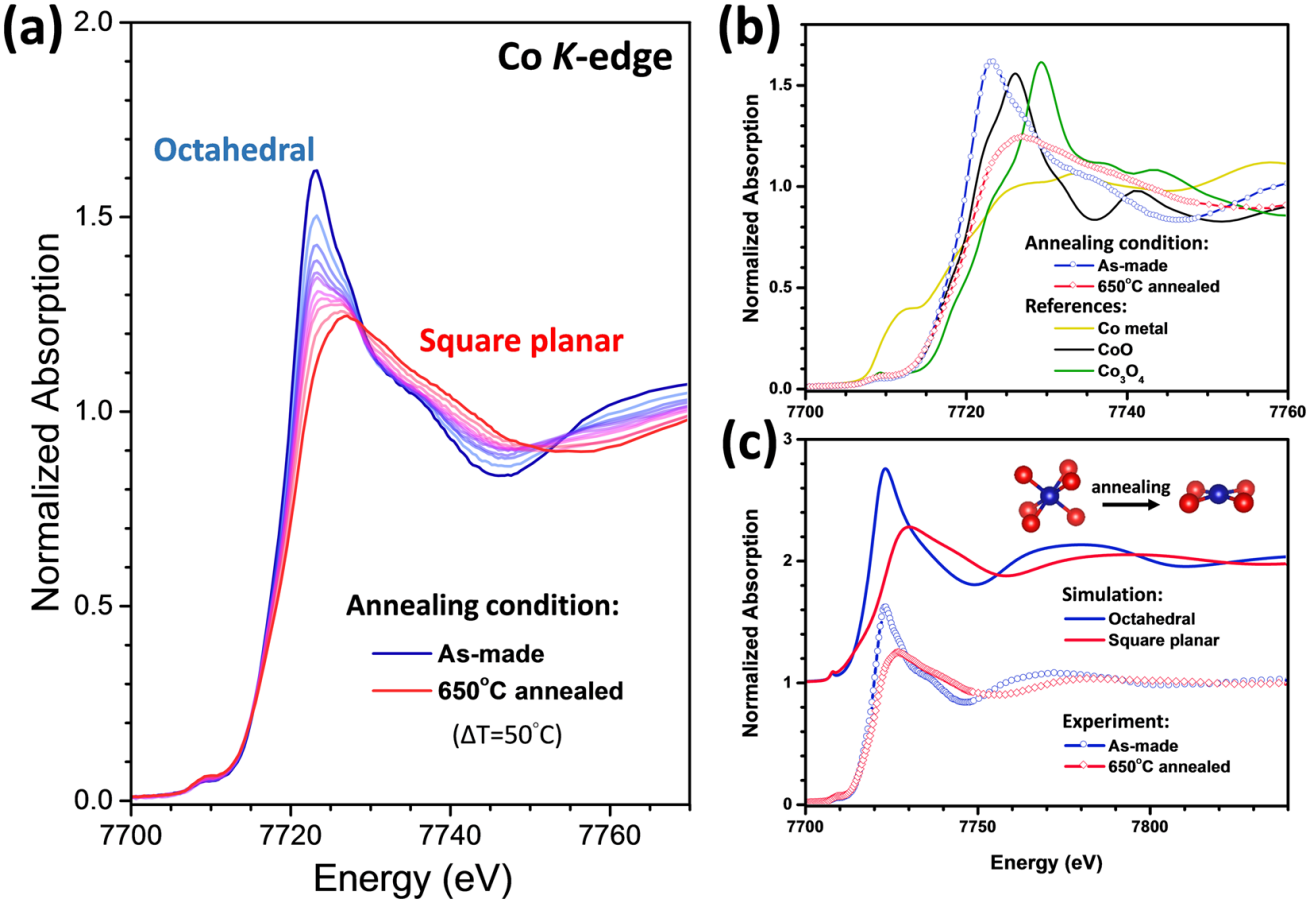
(**a**) Co XANES data for Co-doped CeO_2_ sample annealed at different temperatures. (**b**) Comparison of XANES of as-made and 650 °C-annealed samples with spectra from reference compounds. (**c**) FDMNES simulated Co XANES spectra.

Since the XRD data show no obvious structural changes in the ceria host, possible local structural variations surrounding the Co dopant atoms may play a pivotal role in the mechanism underlying the observed band gap narrowing. To probe the local structure surrounding Co atoms, Co K-edge x-ray absorption near edge structure (XANES) and extended x-ray absorption fine structure (EXAFS) were measured. As shown in Fig. 3(a), the XANES data gradually changes with the increasing annealing temperature. By comparing the XANES of the samples with those of the model compounds, as plotted in Fig. 3(b), the possibility for Co atom forming metal or oxide clusters in the samples can be excluded.

**Figure 4:**
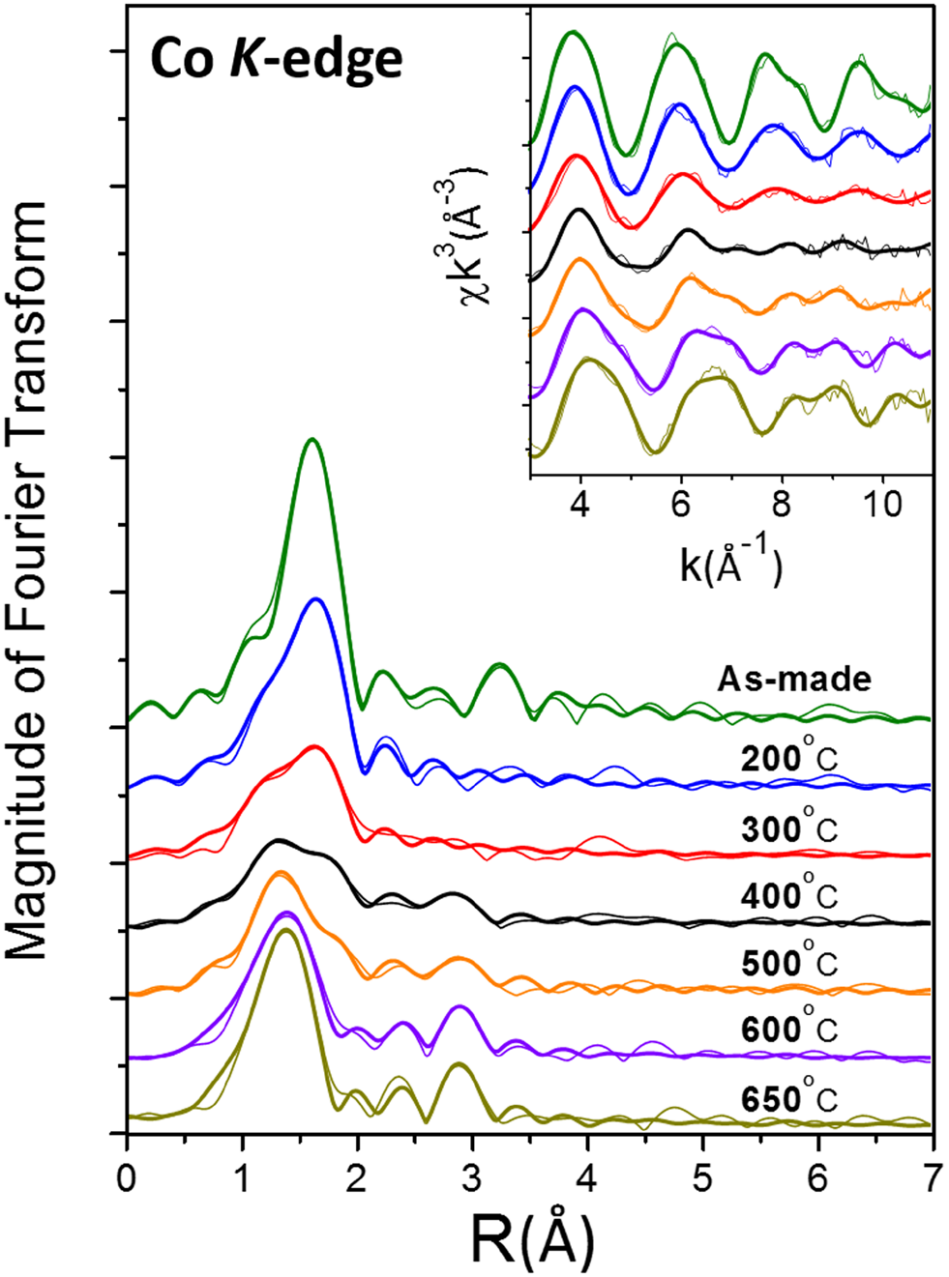
Co K-edge EXAFS data for Co-doped CeO_2_ samples. Fine lines: experimental; Coarse lines: curve fitting. Curves have been shifted vertically for the sake of clarity.

Quantitative information on radial distribution of neighboring atoms surrounding the Co dopant atoms was obtained from EXAFS data. An established data reduction method was used to extract the EXAFS χ-functions from the raw experimental data^17, 18^. The χ-functions of the Co K-edge EXAFS are then Fourier-transformed into real space and plotted as fine lines in Fig. 4. Local structural parameters were quantitatively extracted from the EXAFS functions using an improved curve-fitting procedure with back scattering amplitude and phase shifts functions obtained from the FEFF software^19, 20^. The amplitude reduction factor S_0_^2^ representing the central atom shake-up and shake-off effects and the mean free path of photoelectrons λ were set to be 0.72 and 10 Å as determined in previous papers^21, 22^. The final values of fitting parameters for the Co K-edge EXAFS are listed in Table 1.

**Table 1:** Parameters of local structure around Co atoms obtained from curve-fitting of the Co K-edge EXAFS. N is the coordination number.

Sample	Bond	*N*	*R* (Å)	σ^2^ (10^−3^ Å^2^)	Δ*E*_*0*_ (eV)
As-grown	Co - O	5.7 ± 0.1	2.06 ± 0.01	7.6 ± 0.3	−1.9 ± 0.5
	Co – Ce	2.9 ± 0.4	3.79 ± 0.01	8.1 ± 1.3	−10.1 ± 1.0
200 °C	Co - O	0.5 ± 0.1	1.86	1.3 ± 0.5	−9.1 ± 5.0
	Co - O	4.1 ± 0.2	2.06	6.1 ± 0.8	−2.7 ± 0.5
300 °C	Co - O	1.0 ± 0.1	1.86	5.5 ± 2.3	−10.4 ± 5.0
	Co - O	2.4 ± 0.2	2.06	6.4 ± 1.3	−3.5 ± 0.5
400^°^C	Co - O	1.3 ± 0.1	1.86	6.6 ± 0.5	−7.8 ± 0.5
	Co – O	2.0 ± 0.1	2.06	7.9 ± 0.6	−3.8 ± 0.5
	Co - Ce	1.6 ± 0.2	3.23 ± 0.01	13.9 ± 1.6	−2.0 ± 1.3
500 °C	Co - O	1.5 ± 0.1	1.86	4.2 ± 2.5	−10.2 ± 0.5
	Co – O	1.3 ± 0.1	2.06	3.9 ± 1.3	−2.9 ± 2.5
	Co - Ce	1.2 ± 0.2	3.25 ± 0.02	9.8 ± 2.5	−2.0 ± 1.3
600 °C	Co - O	3.9 ± 0.3	1.87 ± 0.01	13.3 ± 1.4	−9.7 ± 5.0
	Co – Ce	1.3 ± 0.4	3.22 ± 0.02	7.8 ± 2.9	−5.5 ± 5.0
650 °C	Co - O	3.9 ± 0.3	1.86 ± 0.01	10.1 ± 1.1	−8.1 ± 5.0
	Co – Ce	1.4 ± 0.4	3.21 ± 0.02	6.9 ± 3.0	−8.3 ± 5.0

R is the bond length. σ^2^ is the Debye-Waller-like factor serving as a measure of local disorder. ΔE_0_ is the difference between the zero kinetic energy value of the sample and that of the theoretical model used in FEFF. Uncertainties were estimated by the double-minimum residue (2χ^2^) method.

For the as-made sample, the EXAFS data exhibit a nearest O shell at 2.06 Å and a Ce next nearest shell at 3.79 Å from the Co central atom as shown in Table 1. The coordination number of the nearest O neighboring shell is around 6, roughly two less than that of 8 surrounding Ce in the CeO_2_ host. Compared to the 2.34 Å Ce-O distance and 3.83 Å Ce-Ce distance calculated from the standard crystal structure^19^ of CeO_2_, the observed Co-O and Co-Ce distance indicate that the Co dopant atoms most likely substitute for Ce atoms in the CeO_2_ matrix with large distortion arising from the surrounding oxygen vacancies. After thermal annealing, the amplitude of the peak due to the 2.06 Å Co-O bond decreases while a new peak representing a much shorter (1.86 Å) Co-O bond appears and intensifies as the annealing temperature increases. The Co-Ce bond length also decreases from the as-made value of 3.79 Å to 3.22–3.25 Å in the samples annealed at 400–600 °C. For the 650 °C annealed sample, the Co-O and Co-Ce bond lengths are around 1.86 Å and 3.21 Å, respectively. The coordination number of the nearest O neighboring shell is also reduced from 6 to 4. We ascribed this dramatic reduction of Co-O bond length and O coordination number surrounding Co to the change of Co dopant location in the CeO_2_ host due to thermal annealing.

To reveal the coordination geometry around Co atoms, the experimental XANES spectra were compared with theoretical simulation based on the multiple-scattering (MS) formalism using the FDMNES code^20^. Local structural information obtained from EXAFS analysis were used to construct the theoretical model. The self-consistent muffin-tin (MT) full-multiple-scattering (FMS) approach with the real Hedin-Lundqvist exchange-correlation potential was applied with a cluster radius R = 2.1 Å that corresponds to 7 and 5 atoms for the octahedral model and the square planar model, respectively. The FMS calculations were performed using the MT potential constructed from 10% overlapped MT spheres. For the as-made and 650 °C annealed samples, an octahedral and a square planar ligand models with 6 and 4 Co-O bonds were adopted, respectively. As shown in Fig. 3(c), it is clear that the theoretical simulation agrees rather well with the experimental XANES data. Therefore, our x-ray results show that the Co dopant atoms can be driven by thermal annealing from an octahedral coordination in the as-made sample to a square planar coordination.

**Figure 5:**
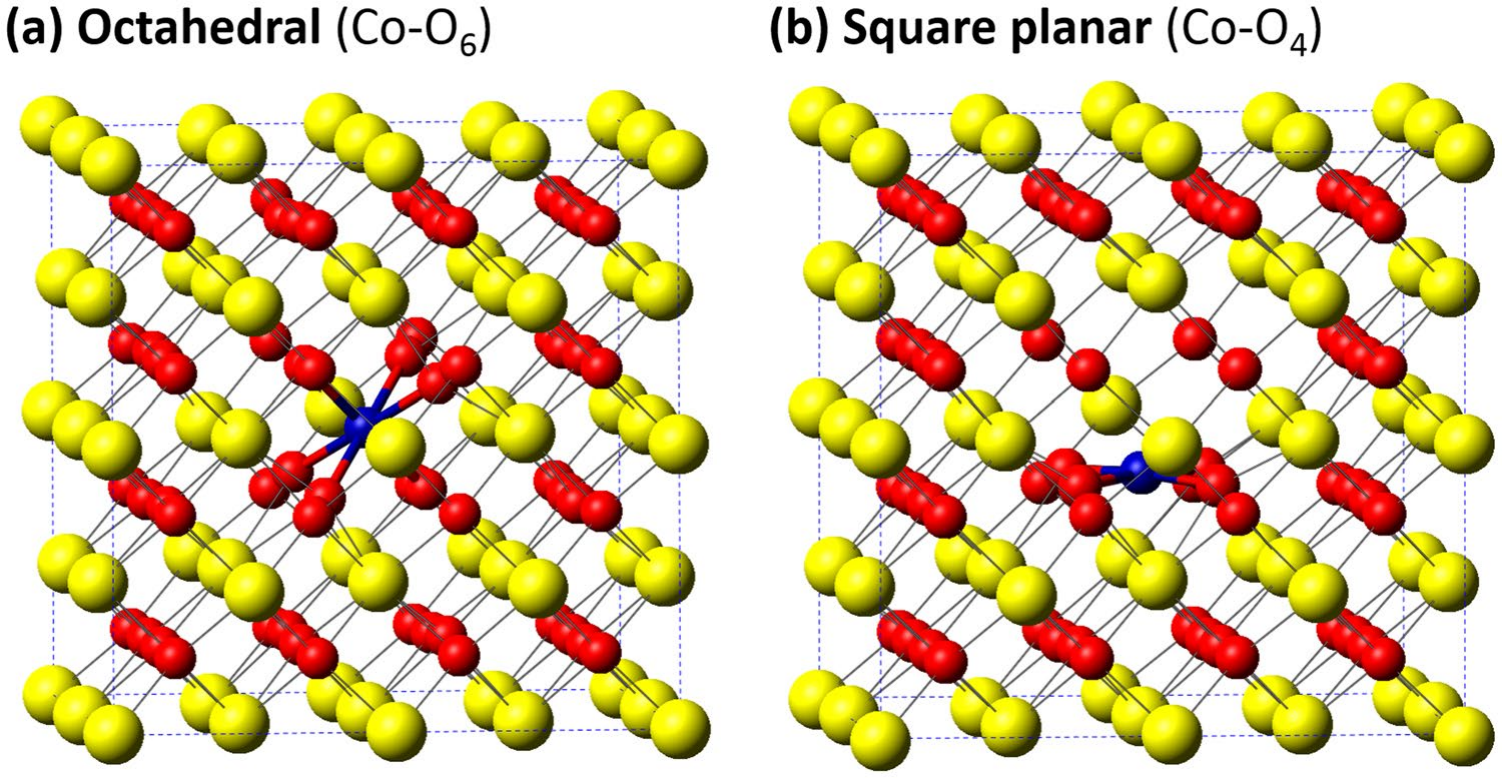
Schematic view of Co doped CeO_2_ with different Co dopant coordination geometry: (**a**) octahedral coordination (**b**) square-planar coordination. The yellow balls, red balls, and blue balls represent the Ce, O, and Co atoms, respectively.

To understand the observed correlation between band gap changes and cobalt local structure transition, density functional theory (DFT) calculations were performed using the Vienna *ab initio* simulation package (VASP)^23^. A generalized gradient approximation (GGA) PBEsol functional^24^ was adopted and a 6.5 eV Hubbard U correction^25–27^ to the Ce 4 f orbitals was employed to describe the correlation effects of the Ce 4 f states in defective CeO_2_ crystals. A 2 × 2 × 2 supercell with 96 atoms therein was used as the initial model for pure CeO_2_. The Brillouin-zone (BZ) integration was performed using a 4 × 4 × 4 Monkhorst- Pack k-point grid^28^. The plane-wave kinetic energy cutoff was set to be 400 eV. Atomic positions and lattice parameters of all models were optimized until the maximum force on each atom was smaller than 0.02 eV/Å. The structural model with octahedrally coordinated Co atoms shown in Fig. 5(a) was constructed by substituting a Ce atom in the ceria host with a Co atom, where two O neighboring atoms were removed from the diagonal sites of a CoO_8_ cube forming an octahedral coordination geometry. In the model with Co dopant atoms in square planar coordination shown in Fig. 5(b), a Co atom was located in the center of the bottom face of a CoO_8_ cube with four O atoms on the top face removed.

After structural relaxation, the calculated lattice constants of octahedral and square planar coordination are 5.400 and 5.426 Å, which are close to the XRD values 5.42 Å. For the octahedral coordination, the calculated average bond length of the nearest O and the next nearest Ce shells around Co are 2.00 Å and 3.81 Å, which are close to the EXAFS values of 2.06 ± 0.01 Å and 3.79 ± 0.01 Å, respectively. For the square planar coordination, the calculated average Co-O and Co-Ce bond lengths are 1.84 Å and 3.24 Å, which satisfactorily reproduce the experimental values of 1.86 ± 0.01 Å and 3.22 ± 0.01 Å, respectively. Both structural models appear to be stable for the Co dopant atoms in the ceria host.

**Figure 6:**
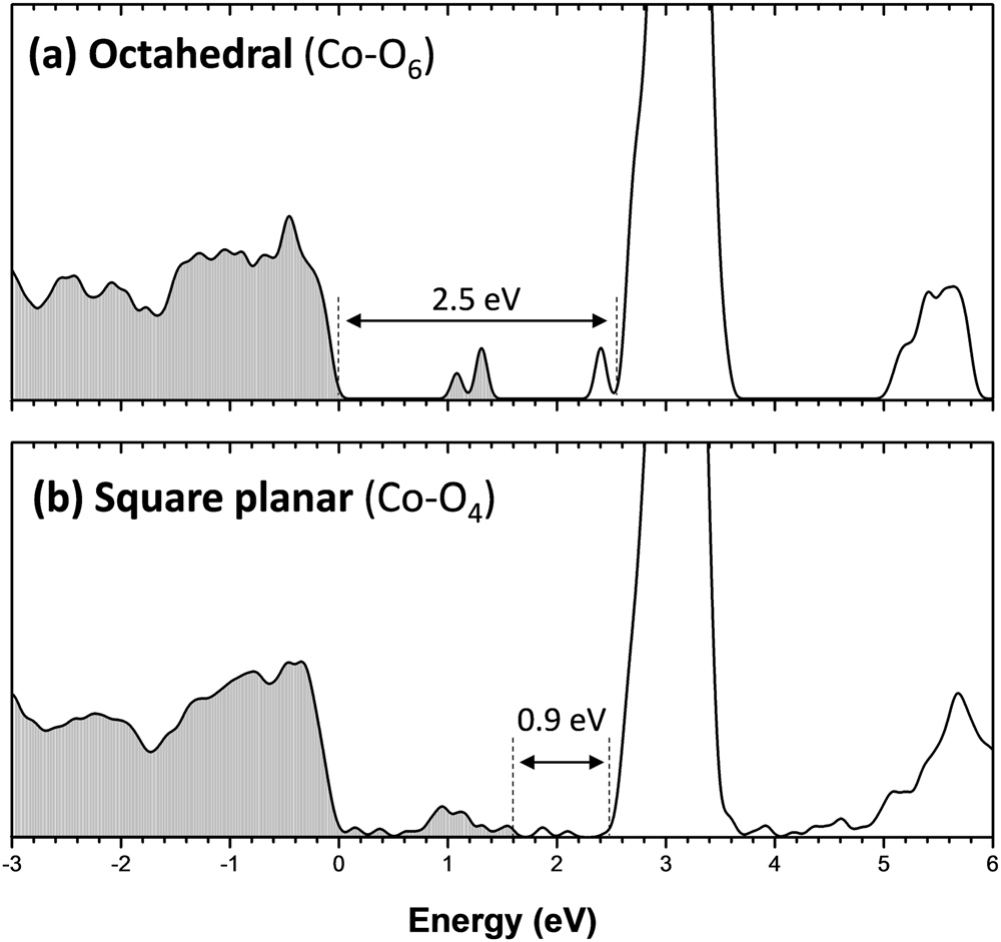
Density of states (DOS) of Co-doped CeO_2_ with different Co dopant coordination geometry: (**a**) octahedral coordination (**b**) square planar coordination.

The calculated densities of states for the octahedral coordination and the square-planar coordination are plotted in Fig. 6(a) and (b), respectively. For the octahedral coordination model, the calculated O 2p to Ce 4 f band gap (2.5 eV) is 0.3 eV smaller than the experimental value of 2.8 eV. This is expected since GGA is well-known to underestimate band gap values. The impurity band isolated in the energy gap is contributed from the Co t_2g_ states and the Ce 4 f^1^ states due to the two O vacancies. For the square planar coordination model, the calculated gap between O 2p and Ce 4f is still 2.5 eV. However, the square planar crystal field incurs more complicated splitting of the Co 3d states. The increased number of oxygen vacancies also introduces more Ce 4f^1^ related states. These impurity states are therefore expanded to connect with the O 2p band such that the band gap width is reduced form 2.5 eV to 0.9 eV. Such a 1.6 eV reduction of band gap width is consistent with the experimentally observed value.

As a side remark, we note that the strain in the nanoparticle can also affect the band gap. However, as the annealing temperature increases, the particle size also increases as indicated by the XRD analysis using Scherrer equation. The crystals of the annealed samples are thus expected to be more bulk-like that should have resulted in a band gap closer to that of the bulk value under strain consideration. However, the band gap value turned out to move rapidly away from the bulk value as the annealing temperature increases. Therefore, the band gap narrowing effect due to coordination rearrangement is apparently dominant over the effect of strain in the present case.

**Figure 7:**
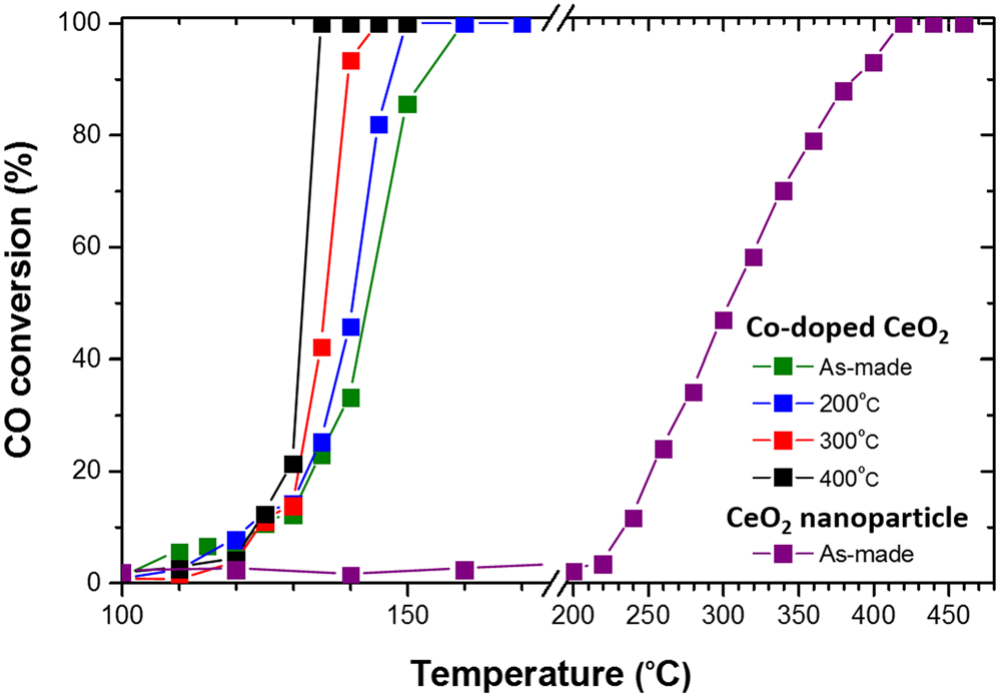
Catalytic activity of CO oxidation for CeO_2_ and Co-doped CeO_2_ nanoparticles annealed at different temperatures.

To explore possible enhancement of catalytic activity due to the observed band-gap narrowing effect in the annealed samples, the activity of each sample as a catalyst in the reaction ${\rm{CO}}+\frac{1}{2}{{\rm{O}}}_{2}\to {{\rm{CO}}}_{2}$ was monitored by quantifying the concentration of the effluent gas with a gas chromatograph (GC) device equipped with a thermal conductivity detector. The catalyst weight was 30 mg and the total flow rate of the reaction gas was 100 SCCM, with a composition of 5% CO– 25% O_2_ (balanced with He gas). The conversion of CO was calculated from the CO concentrations in the inlet and outlet gases. It is clear that the sample becomes more active with the cobalt dopant, as shown in Fig. 7. The light-off temperature T_50_, corresponding to 50% conversion of CO, for the as-made Co-doped sample around 143 °C is much lower than that for the CeO_2_ nanoparticle sample of 305 °C. On the other hand, the catalytic activity also shows progressive enhancement as the band gap decreases with increasing annealing temperature up to 400 °C. We note that the nanoparticle sizes for the 200 °C-, 300 °C-, and 400 °C -annealed samples are very close to that for the as-made sample. When the annealing temperature increases beyond 400 °C, the nanoparticle size increases abruptly and the change of catalytic activity no longer follow the trend, due to the dominating counter effect of large particle size.

In conclusion, we have observed dramatic band gap reduction in thermally annealed samples of Co-doped CeO_2_ nanocrystal catalysts. Experimental and theoretical analyses indicated that these effects are due to local structural transition of Co from an octahedral coordination to a square-planar coordination. We have demonstrated a mechanism of a totally different concept for band gap reduction in doped nanocrystal materials. Dramatic reduction of band gap due to dopant coordination rearrangement incurred by thermal annealing in nanocrystals of Co-doped ceria is of great potential for applications in a wide variety of energy and environmental technologies.
